# A Review of Woodhull’s Non-Emetic Use of Ipecacuanha

**Published:** 1877-01

**Authors:** Ely McClellan


### A REVIEW OF WOODHULL'S NON-EMETIC USE OF IPECACUANHA.

Studies Chiefly Clinical, in the Non-Emetic Use of Ipecacuanha; with a Contribution to the Therapeusis of Cholera. By Alfred A. Woodhull, M.D., Assistant Surgeon and Brevet Lieut. Col. U. S. Army. Philadelphia: J. B. Lippincott & Co. 1875. 8-vo. pp. 155.

But it is well remarked, in Plato's Limœus, that vomits and purges are the worst exercise in the world.—*Berkeley.*

When the improbability of a fact is the chief objection to the belief in its reality, the evidence which attests it requires all its value, if the impossibility be proved only apparent.—*Salverte.*

Because the studies of Woodhull in the non-emetic use of Ipecacuanha first saw light in the pages of the ATLANTA MEDICAL AND SURGICAL JOURNAL; because he was successful in causing an enthusiasm among some of our brethren in this State; and finally, because so much earnest and conscientious work has been expended upon the subject, that a revival in the employment of a long neglected drug has, by this effort, been brought about, we propose to present to its readers a rather more elaborate resume of the work than is usually devoted to productions of like character.

What could be more auspicious than the extracts from Berkeley and Salverte, which have been selected as the legend of the title page. The mental association between ipecac. and and vomits is so strong that it is with great difficulty that even an educated mind can be brought to view the Brazilian root from any other standpoint. The theory advanced is not based upon improbable conjectures. They are proved realities, by which the hypothesis that ipecacuanha, exhibited in other than very small doses, vomits, is exploded; and the demonstration is attempted, that its therapeutic action is that of a direct nervous stimulant, which acts chiefly, if not entirely, upon the sympathetic nervous system.

The work is an outgrowth of an attempt to solve this therapeutic problem. It has passed through these stages of development: I. A series of reports to the Surgeon General of the U. S. Army. II. A series of papers in this journal (from Feb. to July, 1875). III. The volume before us, which is the former work elaborated. The study is divided into three parts: I. Clinical facts. II. Therapeutic opinions. III. A speculation upon cholera. In part I. the following arrangement of subjects is observed: *Acute Dysentery, Chronic Dysentery and Chronic Diarrhœa, Enemata af Ipecacuanha, Painful but Simple Intestinal Affections, Cholera Morbus and Cholera Infantum, Uterine and other Hemorrhages, Excessive Perspiration, Dyspepsia, Vomiting of Pregnancy, Asthma and Nervous Coughs, Drunkenness and Delirium Tremens, Opium Poisoning, Neuralgia, Intermittent Fever, Febrifuge Action, Pneumonia, the Puerperal State, Hepatitis, Antidote to Venom, and Topical effects.*

At first sight, upon observing the great diversity of affections in which the drug is recommended, one is inclined to discredit the assertions of the author upon page 125, "that it is not a special plea for that drug to the exclusion of others." But it must be remembered that the therapeutic virtues claimed are those of a direct stimulant to the sympathetic nervous system; and when we recall the fact, as stated by Flint, that, "according to the latest researches, the filaments of the sympathetic, at or near their termination, are connected in ganglionic cells, not only in the heart and the uterus, but in the blood-vessels, lymphatics, the sub-mucous and the muscular layer of the entire alimentary canal, the salivary glands, liver, pancreas, larynx, trachea, pulmonary tissue, bladder, ureters, the entire generative apparatus, supra-renal capsules, thymus, lachrymal canals, ciliary muscle, and the iris* we are prepared to acknowledge that in each and every one of the dis-

* Physiology of Man, Nervous System. Flint, p. 424.

cases named, should ipecacuanha be proved to be a stimulant to the sympathetic system, it may be ranked as a remedial agent worthy of the closest study.

In part II. the peculiar therapeutic operation of ipecacuanha is discussed as regards the diseases already enumerated, with a disquisition upon its neurotic properties, and a discussion of the nature of dysentery, with opinions as to the reasons for and the manner of the use of the drug in that disease.

It would be impracticable to follow Dr. Woodhull through the pages of his work, although each presents suggestions and thoughts worthy of the most careful consideration, while many are marvels of specious reasoning. The key note of this work is the supposed therapeutic action of ipecacuanha upon the sympathetic nervous system, and its well-known action in dysentery is brought forward as a ready illustration. Years since the drug earned the empirical title of Radix Dysenterica. The rationale of its action has been a vexed question. Writers have indulged in a vast amount of reasoning upon the subject, but as yet no definite conclusion has been reached. Woodhull in bringing forward the sympathetic nervous system, advances a rather more plausible theory than those before him, though as yet it is but a pure speculation. He announces his belief that dysentery, "at its inception, is the manifestation of a peculiar ganglionic intoxication; that the intestinal inflammation, with its consecutive ulceration, is a result of the malady, but not the primary or radical affection." The practical application of which theory is as follows: "The first step in the disease is a relaxation of the ordinary nervous tone of the part; the capillaries released from nerve control, immediately leak; the glands, with an unbalanced nerve supply, fail either in the quality or the amount of their secretions; the blood supply increases and stagnates in the vascular maze, soon transforming it into a wilderness whose neo-plastic growths, like tares and brambles, add to the desolation. But if at any time before the structural changes set in (which is the point that I look upon as the commencement of inflammation), the nerve control is reinstated, we then find the congestion renewed, the glands properly fulfilling their offices, the capillaries no longer exuding blood and serum, and, *pari passu*, the abnormal sensations disappearing. That is, the process whose continuance would have developed inflammation is abruptly altered. And it is a main object of this paper to show that ipecacuanha may exert just such control. That simple view of the matter seems to me to entirely explain the incidents. Later in the disease, when plastic changes have occurred, we must suppose that the nervous depression persists and aggravates them, so that to the very last the indication is to neutralize it in the hope that, the proper balance being established, the restorative power of nature will gradually lift the patient to the line of health, permitting the repair of the ulceration and the absorption of the extraneous matter. But it is altogether probable that the condition of ulceration is not nearly so persistent in either form of dysentery as is generally supposed." pp. 88--89.

Assuming that dysentery, save in scorbutic diarrhœa, is rarely, if ever, caused by indigestible ingesta, and omitting all consideration of the contagion hypothesis, the poison of malaria is announced as a prolific source of the disease—"the common though probably not the sole cause."

It does seem, that as progressive, reasoning beings, we should look for causes beyond the wonderful, impalpable something dubbed malaria. Malaria, an imponderable force developed by organic decomposition. Malaria, an atonic force developed under certain conditions of vegetable decomposition (p. 110), are the contributions of the author to the many definitions of this agent, and ingeniously speculative they are. But is it not wisdom to include dysentery among the acute infectious diseases? Does not the natural history of the disease point more clearly to that than to the powers of malaria?

To return to our subject. It is therefore concluded that ipecacuanha being a nervous stimulant, acting chiefly through the sympathetic system, is capable, when exhibited in sufficient doses, of re-establishing the nerve control by which this congestion is removed; the glands required to resume their normal functions, and the capillaries to retain the *blood and serum* flowing through them.

This hypothesis is based entirely upon the theory of impeded nervous control. But does even section of the sympathetic nerves induce excessive hyperæmia of a serous effusion within the intestines? Accepting the experiments of Moreau, published in 1850, as final, we would be compelled to answer,

Yes. It is a fact, however, that they remain without verification at the hands of other observers. In 1872, the experiments of Drs. T. R. Lewis and D. D. Cunningham were published, wherein were recounted a series of eight experiments upon the splanchnic and sixteen upon the mesenteric nerves, in which, save in one instance, opposing results were obtained.

The evidence, as yet, is far from positive. The fact that ipecacuanha exerts a specific influence upon dysentery is fully established, and the reasoning of Woodhull is certainly plausible; more so than that of the vast majority of those who have preceded him. It will undoubtedly bear fruit in due season.

The respective influences of malaria in causing, and of ipecacuanha in removing dysenteries, having been fully established in the mind of the author, the transition to intermittents is over an easy grade. If we can accept the logic advanced, the point is proved.

The scene changes. The same cause operating upon the sympathetic nervous system, which formerly induced dysentery, now causes intermittents (p. 110); and later the identical cause will produce cholera (p. 128). The broad distinction which epidemiologists have adopted of Cholera Asiatica must be abandoned, and if this theory is to be accepted, we will have Russian, German, French, English, and American cholera. How strangely this sympathetic of ours does act: at one time loosing its grip upon the intestines; the capillaries leak blood and and serum (p. 88). Again, the identical cause permits the leakage from the intestinal capillaries of pure serum—rice water. In one case the solid constituents of the blood are discharged, in the other but the fluid; the solids remaining within the vessels, plug them with the thick, tenacious mass to which the blood has been transformed by the process of dehydration.

It is useless to follow this further. That ipecacuanha is a drug of extreme usefulness is fully established, and Dr. Woodhull deserves great credit for the care with which he has collected such a mass of evidence within so reasonable a compass. A most signal service has been rendered to mankind in this earnest revival of ipecacuanha. The volume claims, and should be accorded, a prominent place upon the shelves of all medical men. While the majority of modern students cannot accept all the hypotheses advanced, yet it will repay close and constant study.

